# Metabolic and Anthropometric Influences on Nerve Conduction Parameters in Patients with Peripheral Neuropathy: A Retrospective Chart Analysis

**DOI:** 10.3390/neurolint13020016

**Published:** 2021-04-15

**Authors:** Daniel H M Ly, Venkat N. Vangaveti, Ravindra Urkude, Erik Biros, Usman H Malabu

**Affiliations:** 1 Translational Research on Endocrinology and Diabetes (TREAD), College of Medicine and Dentistry, James Cook University, Douglas, QLD 4811, Australia; mynameis.daniel@yahoo.com (D.H.M.L.); venkat.vangaveti@jcu.edu.au (V.N.V.); erik.biros@jcu.edu.au (E.B.); 2 Department of Neurology, Townsville University Hospital, 100 Angus Smith Drive, Douglas, QLD 4814, Australia; ravindra.urkude@health.qld.gov.au

**Keywords:** nerve conduction study, peripheral neuropathy, body mass index, anthropometry

## Abstract

Background and Aims: Nerve conduction study (NCS) measures how fast an electrical impulse moves through the nerve and is a standard technique for diagnosing and assessing neurological diseases. Despite diabetes and obesity being a common accompaniment of peripheral neuropathy, their effects on NCS patterns have not been elucidated conclusively. Our study aimed to assess several anthropometric and metabolic factors with NCS outcomes to address this gap. Research Design and Methods: This retrospective chart analysis study was conducted on subjects who underwent NCS between 1 January 2009 and 31 December 2019 at a regional hospital. Metabolic, anthropometric, demographical and NCS data were collected from patients’ health records. Results: In total, 120 subjects presenting with sensorimotor peripheral neuropathy symptoms were included in the study. Age, HbA1c, urea and ESR variables were significantly negatively associated with nerve conduction outcomes (Spearman’s correlation rho between −0.210 and −0.456, *p* < 0.038). HbA1c and age consistently had the most substantial contribution to velocity and amplitude in all regression models (beta coefficients between −0.157 and 0.516, *p* < 0.001). Urea also significantly account for a large amount of variance in amplitude and velocity in the lower limbs. Conclusion: This study suggests that the severity of sensorimotor neuropathy is influenced by glycaemic control, age and uraemia. The interpretation of NCS results must consider these factors suggesting that improved glycaemic and uraemic control may improve nerve conduction outcomes.

## 1. Introduction

Peripheral neuropathy refers to the disturbance in the somatic nervous system’s sensory and motor function associated with the skeletal muscle control of body movements and sensation. It is a relatively common neurological disorder that is heterogeneous in aetiology. It is estimated that peripheral neuropathy affects almost 9% of the general population, with prevalence increasing with age [1,2]. When undiagnosed or undermanaged, the physical, psychosocial and financial consequences may potentially be debilitating to the patient [3,4].

The diagnosis of sensorimotor peripheral neuropathy requires a careful history and clinical examination. Nerve conduction study (NCS) is commonly employed as an adjunct when the diagnosis remains uncertain. Testing helps to localize the lesion site, particularly identifying whether the peripheral nerve, neuromuscular junction or anterior horn cells are involved. More importantly, it also helps to differentiate demyelination from axonal loss [5]. The parameters measured and used for interpretation include nerve conduction velocity, amplitude and onset, peak and F-Wave latencies [5]. However, the two most important NCS parameters in the interpretation, thus the focus of this study, are velocity and amplitude.

Both amplitude and velocity are affected by several risk factors contributing to the pathogenesis of neuropathy. Anthropometric and metabolic factors, including age, temperature and height, have all been demonstrated in some capacity to correlate with NCS parameters [6,7,8,9] directly. Furthermore, several physical and metabolic parameters require correction or allowance when performing or interpreting the NCS results. Hemoglobin A1c (HbA1c) and body mass index (BMI) have also been investigated in univariate models [6,7,8,9,10], but multivariate techniques have only been used by one study without taking into account metabolic risk factors [8]. Besides this, few other metabolic markers such as hypertension and dyslipidemia have been investigated for their effects on the NCS outcomes [10,11].

The present study aimed to assess the association of anthropometric and metabolic risk factors with NCS and identify risk factors most significantly influencing the NCS outcomes and severity of neuropathy.

## 2. Materials and Methods

### 2.1. Research Design and Population

This study was a retrospective chart analysis of neurology clinic patients undergoing NCS for suspected peripheral neuropathy at the Townsville University Hospital, Australia. Ethics approval was obtained (HREC/14/QTHS/128). Included were patients who underwent NCS from 1 January 2009 to 31 December 2019. Motor and sensory NCS of lower and upper limbs were conducted based on the symptoms’ site in consenting patients. Anthropometric variables included: age, weight, height and BMI. Metabolic variables studied included: HbA1c, erythrocyte sedimentation rate (ESR), thyroid-stimulating hormone (TSH) and urea. Nerves studied included the motor median, ulnar, tibial, superficial peroneal and sensory median, ulnar, and sural and superficial peroneal nerves. Patients were excluded if: under 18, they had greater than two missing metabolic or anthropometric variables on drugs known to cause peripheral neuropathy or had solitary upper/lower limb studies.

### 2.2. Procedural Details and Data Collection

The NCS was performed using a Nicolet Viking Quest (Nicolet Biomedical, Madison, WI, USA) by the same technician. The temperature was maintained at 32° Celsius in both upper and lower limb NCS. For motor NCS of the upper limb, both ulnar and median nerves were stimulated at the wrist, located approximately 6.5 cm proximal to the recording electrode. Ulnar and median sensory responses were obtained using digits V and II, 11 cm and 13 cm proximal to the recording electrode, respectively. The motor component was stimulated at the ankle’s level in the lower limbs, at 8 cm proximal to the recording electrode in both superficial peroneal and tibial motor studies. Sensory response in the lower limbs was obtained at the lateral aspect of the ankle for the superficial peroneal nerve, at 12 cm proximal to the recording electrode. If NCS was conducted unilaterally, then that sole value was used. A majority (93%) of the study population had bilateral NCS, and for these cases the average velocity, amplitude and latencies of both sides were calculated. In the event of an absent reading in a measured nerve, conduction velocity and amplitude were labelled as “0.00”, and latency entered as a missing value.

Similarly, these values were labelled and entered as a missing value in all unmeasured nerves and omitted from statistical analysis. Two subjects with nerve conduction velocities greater than 100 ms^−1^ were excluded from the analysis due to values greater than 100 ms^−1^ that were considered aberrant recordings.

Patients undergoing the NCS were seen in consultation before the study, where they were asked for a blood sample to measure their metabolic parameters. The specific investigations depended on the patient’s presentation and comorbidities. Anthropometric and metabolic variables collected pertinent to this study included age, sex, height, weight, BMI and HbA1c, TSH, urea, and ESR, respectively. Each chosen independent variable represents a measurement of a potential underlying risk factor for sensorimotor neuropathy. Diabetes was diagnosed if fasting blood glucose level > 7 mmol/L or random blood glucose level > 11.1 mmol/L or HbA1c > 6.5% or patient was on treatment for diabetes. Anthropometric data were obtained by reviewing patient charts, and metabolic data were obtained through the pathology database AUSLAB. ESR, urea, and TSH were used if within one month of NCS recording. Blood samples were collected and biochemical parameters were analyzed within one month of the NCS. HbA1c, age, height and weight were permissible if data were obtained within three months of the NCS.

### 2.3. Statistical Analysis

The analysis was performed using SPSS version 23.0 (IBM Corp. Released 2015. IBM SPSS Statistics for Windows, Version 23.0. IBM Corp, Armonk, NY, USA). Nerve parameters were first summated by type (sensory or motor), parameter (velocity or amplitude) and location (upper or lower limbs) and transformed into a composite measure. These composite measures were the dependent variables reflecting the severity of neuropathy (Table 1). The data were then tested for normality using the Shapiro–Wilk test. Spearman’s Rank Correlation between the aforementioned composite groups and independent variables was calculated in univariate analysis. Independent variables found to have a linear relationship in univariate analysis were subsequently included in multiple regression models. Standard multiple linear regression was used in multivariate analysis to account for and evaluate the individual effects of independent variables on nerve conduction parameters. All tests were two-tailed, and a *p*-value of <0.05 was considered to be significant. Results were expressed as three significant figures. Post hoc validation of the multiple regression models was done using the Durbin–Watson statistic. Bonferroni correction for multiple testing was also done to address potential type 1 errors.

## 3. Results

### 3.1. Baseline Cohort Demographics

After chart screening, a total of 120 participants were included in the statistical analysis. The median age was 62, and 54% of our participants were males. 73% of the participants were diabetic. All NCS measurements for our study population were >40 m/s and none was <30 m/s. The baseline demographics are shown in Table 2.

### 3.2. Univariate Correlation

The results of the univariate correlation are shown in Table 3. Linear relationships were found to best explain the data in all cases. A moderately inverse correlation was demonstrated between the sum of motor nerve velocities (SMNV) and the sum of sensory nerve velocities (SSNV) with age, ESR, HbA1c and urea. These results were also statistically significant. Spearman’s correlation coefficients ranged from −0.217 to −0.363 (Table 3). The sum of motor nerve amplitudes (SMNA) and the sum of sensory nerve amplitudes (SSNA) were similarly inversely correlated with age, ESR, HbA1c and urea (Table 3). TSH also demonstrated the same linear, inverse correlation, but sometimes in the absence of significance (rho between −0.106 and −0.214; Table 3). BMI correlated with neither amplitude nor velocity (Table 3).

Similar trends were found when nerve conduction outcomes were divided based on location (upper and lower limbs). Age, ESR, HbA1c and urea all significantly demonstrated a moderate, inverse correlation to nerve conduction velocity and amplitude (rho between −0.162 and −0.415) in both upper and lower limbs. TSH consistently demonstrated the same trends but in the absence of significance. Again, BMI did not correlate with velocity and amplitude.

### 3.3. Multivariate Correlation

The same variables identified in univariate analysis were included and studied in multiple regression analysis except for BMI (Table 4 and Table 5). This variable was omitted from inclusion into multiple regression models given a lack of real linear relationship in univariate analysis.

When disregarding the nerve’s location, these models accounted for 18.3% to 38.6% of the nerve conduction variance. In both motor and sensory nerve amplitudes, age was found to have the strongest unique contribution (beta coefficients = −0.250, *p* = 0.04 and −0.516, *p* < 0.001, respectively) with significance. In models predicting velocity, HbA1c was the strongest contributor in both motor and sensory nerves (beta = 0.356, *p* = 0.001, and 0.277, *p* = 0.027, respectively; Table 4). Urea and TSH had less individual contribution (beta coefficient between −0.064 and −0.319; Table 4) than age and HbA1c. ESR was found to have the least unique contribution (Table 4).

When the nerve location was taken into account (upper/lower limbs), only some trends remained the same. HbA1c continued to yield the most significant contribution to predicting nerve conduction amplitudes and velocities in all limbs. Age was also responsible for a large variance in amplitude and velocity; however, some models were less significant. Both HbA1c and age were inversely correlated to nerve conduction parameters. ESR was also included in multiple regression analysis, but its contributions were negligible and not at all significant (beta coefficient between −0.124 and −0.071; Table 5). TSH did not significantly contribute to the prediction of nerve conduction outcomes in either upper or lower limbs. Urea was also shown to correlate with both nerve conduction amplitude and velocity inversely. Compellingly, in the lower limbs, urea significantly accounted for more sensorimotor variability (beta coefficient between −0.221 and −0.398; Table 5) than both age and HbA1c. However, urea’s upper limb contribution was much less in both domains (beta coefficient between −0.0419 and −0.107; Table 5).

## 4. Discussion

In this present study, HbA1c and age were demonstrated to significantly correlate and impact nerve conduction amplitude and velocity. Urea, ESR and TSH were also shown to inversely correlate with both nerve conduction parameters, albeit to a lesser extent. BMI was not found to correlate with nerve conduction parameters.

As most of our study population had bilateral NCS, the neurophysiological parameters were presented as sums and not for each nerve separately. Interestingly, the decline in velocity was most marked in subjects with higher HbA1c in our sensory and motor assessments. In particular, HbA1c consistently exhibited the most remarkable individual contribution to the decline of nerve conduction velocity in motor and sensory models in both upper and lower limbs. This result is in keeping with the theory that poor glucose control is associated with nondiscriminative demyelinating pathogenesis. Huang et al. and Tkac et al. also showed a decrease in velocity with high HbA1c [6,8]. HbA1c was also inversely correlated to amplitude; however, it accounted for less variance than velocity, similarly documented in other studies [6].

Conversely, age demonstrated the opposite effect. Older age accounted for the most significant amount of decline in the amplitude. However, it was second to HbA1c in contributing to the variance in velocity—consistent with the conclusions of Jagga et al. [11]. This is most likely attributable to age-related axonal degeneration and loss manifesting predominantly as a reduction in amplitude in NCS. This phenomenon is also well-demonstrated in studies by Stetson et al. and Rivner et al., which showed a steady decline in amplitude with each successive decade of life [12,13]. Although the effects of age and glycaemic control on nerve conduction outcomes are well-established, this study extends this knowledge using the multivariate linear analysis to discern how much individual influence each risk factor has on outcomes. Our findings suggest that an adequate glycaemic control corresponds with prevention or improvement on nerve conduction velocity and amplitude in both motor and sensory domains. Age, albeit an inevitable risk factor for nerve degeneration, is also essential to consider when interpreting NCS results based on our results. Aberrant findings in amplitude and velocity will be missed in younger patients if typical values for older subjects are used. Exact reference ranges for velocity and amplitude should be modelled against findings from larger-powered studies such as Rivner et al. and Stetson et al. and thus incorporated into clinical interpretation [12,13]. Urea, a lesser-studied metabolic parameter, also demonstrated an inverse correlation to nerve conduction velocity and amplitude. Our findings show that urea inversely correlated with both nerve conduction velocity and amplitude in upper, lower and combined models. These correlations were most pronounced in the lower limbs’ motor nerves (peroneal and tibial nerves). This finding strengthens the observations noted in previous studies on NCS [14,15,16,17]. Additionally, our study adds to existing knowledge using multiple regression analysis to demonstrate a stark contrast in upper limb results compared to lower limb studies consistent with other findings [18,19].

Similarly, in univariate analysis, ESR was found to have an inverse, linear correlation with sensorimotor velocity and amplitudes in upper, lower limbs and combined models with statistical significance. This result is intriguing, given that an elevated ESR could result from an amalgamation of processes. These may include diabetes, collagen vascular disease, and CKD, all related to sensorimotor neuropathy development [20]. Despite univariate analyses being initially optimistic, once the effects of the other independent variables were accounted for, ESR’s contributions were almost negligible. While a linear correlation exists, this study’s findings suggest the use of ESR as an indicator of peripheral nerve function is limited, given its inconsequential contribution seen in multivariate analyses.

Several studies on the influence of subclinical hypothyroidism on peripheral nerve functions have had mixed results finding significant differences in nerve conduction parameters between subjects with subclinical hypothyroidism and those without [21,22]. Our results could not confirm any correlation between TSH and nerve conduction outcomes. These findings appear to support the premise that there is only a weak correlation between TSH and nerve conduction parameters, as evidenced by Us et al. and Kececi et al. [23,24]. We have shown that the role of TSH has a minor contribution to overall nerve parameters. Given the disparities of conclusions in the literature, further high-powered studies are required to elucidate whether such a correlation exists.

On the other hand, our results found BMI not to correlate with nerve conduction parameters. Neither statistically significant results nor demonstrable relationships between nerve conduction amplitude and velocity were discerned. Numerous studies have also failed to identify a relationship between BMI and nerve conduction parameters. [10,25]

Notwithstanding, the retrospective design of this study presents some limitations inherent with such an approach. Firstly, there was a discrepancy between the timing of the blood analysis and the timing of the NCS. Inclusion criteria permitted the use of blood tests from a maximum of one month before or after the NCS or a maximum of three months for anthropometry and HbA1c. Given that some metabolic parameters have a dynamic nature, this potential gap between blood analysis and the NCS testing time introduces discrepancies. The longer the period between blood analysis and NCS, the less representative the data is.

Consequently, prospective studies would be preferable in preventing this bias. Secondly, we had no control over the selection of blood tests of subjects. Depending on their presentation to clinics, comorbidities and risk profile, some blood tests could have been omitted. Despite our best efforts to cover the most commonly ordered metabolic parameters in the study, a lack of protocol regarding this issue exposes an inherent sampling bias due to our study’s retrospective nature. Thus, our study manifested in several subjects with missing values. Subsequently, subjects with more than two missing metabolic values were excluded from inclusion to reduce this bias. Despite these limitations, the results from our study substantiated that of existing literature. It also extends this by showing that in more comprehensive analysis such as multivariate regression, the overall severity of NCS was most related to age, HbA1c and uraemia.

## 5. Conclusions

The use of NCS must take into consideration patient factors that may influence outcomes. This study shows the significant individual contributions of age, HbA1c and urea on nerve conduction amplitude and velocity. Other metabolic factors, such as TSH and ESR, were shown to have a lesser impact. Interestingly, BMI did not influence these outcomes. Effective glycaemic and uraemic control are, therefore, suggested to have beneficial effects on NCS. Although unamenable to change, the effect of age on nerve conduction parameters should also be taken into account.

## Figures and Tables

**Table 1:** List of composite dependent variables.

Composite Variables	Nerves Measured
SMNV—Sum of Motor Nerve Velocities	Motor: Median + Ulnar + Tibial Nerve velocities
SMNA—Sum of Motor Nerve Amplitudes	Motor: Median + Ulnar + Tibial Nerve amplitudes
SSNV—Sum of Sensory Nerve Velocities	Sensory: Median + Ulnar + Sural Nerve velocities
SSNA—Sum of Sensory Nerve Amplitudes	Sensory: Median + Ulnar + Sural Nerve amplitudes
SULSA—Sum of Upper Limb Sensory Amplitudes	Sensory: Median + Ulnar Nerve amplitudes
SULSV—Sum of Upper Limb Sensory Velocities	Sensory: Median + Ulnar Nerve velocities
SULMA—Sum of Upper Limb Motor Amplitudes	Motor: Median + Ulnar Nerve amplitudes
SULMV—Sum of Upper Limb Motor Velocities	Motor: Median + Ulnar Nerve velocities
SULSMV—Sum of Upper Limb Sensorimotor Velocities	Sensory + Motor: Median+ Ulnar Nerve velocities
SLLMA—Sum of Lower Limb Motor Amplitudes	Motor: Tibial + Superficial Peroneal Nerve amplitudes
SLLMV—Sum of Lower Limb Motor Velocities	Motor: Tibial + Superficial Peroneal Nerve velocities
SLLSMV—Sum of Lower Limb Sensorimotor Velocities	Sensory: Sural + Motor: Tibial + Superficial Peroneal Nerve velocities

**Table 2:** Baseline characteristics of subjects.

Variables (n = 120)	
Sex (Male/Female, % Male)	(65/55, 54.17%)
Age (yr) (n = 120)	62.00, (48.00–68.00)
Height (m) (n = 120)	1.71, (1.64–1.78)
Weight (kg) (n = 120)	77.55, (68.08–93.45)
BMI (kg/m^2^) (n = 120)Overweight/Obese (n = 82)	27.60, (23.61–31.30) 68%
Diabetic HbA1c (%)(n = 87) Duration of DM (years)	6.20, (5.50–7.60) 9 (2–17)
Urea (mmol/L) (n = 117)	5.50, (4.35–9.00)
ESR (mm/hr) (n = 96)	12.00, (8.00–30.75)
TSH (mIU/L) (n = 111)	1.40, (1.00–2.08)
Vit B12 (ng/mL)	238 (182–348)
Chronic Kidney Disease (n = 16)	14.0%
Sum of Motor Nerve Velocities	184 (170–200)
Sum of Motor Nerve Amplitudes	25 (20–33)
Sum of Sensory Nerve Velocities	141 (96–163)
Sum of Sensory Nerve Amplitudes	37 (17–66)
Sum of Upper Limb Sensory Nerve Amplitudes	32 (16–59)
Sum of Upper Limb Sensory Nerve Velocities	98 (84–111)
Sum of Upper Limb Motor Nerve Amplitudes	16 (13–18)
Sum of Upper Limb Motor Nerve Velocities	104 (98–112)
Sum of Upper Limb Sensorimotor Nerve Velocity	202 (183–222)
Sum of Lower Limb Motor Nerve Amplitudes	10 (6–16)
Sum of Lower Limb Motor Nerve Velocities	80 (73–88)
Sum of Lower Limb Sensorimotor Nerve Velocity	123 (81–138)

Data presented as median, (25th percentile-75th percentile).

**Table 3:** Spearman’s correlation coefficients between nerve conduction parameters and metabolic, anthropometric variables.

	SMNV	SMNA	SSNV	SSNA	SULSA	SULSV	SULMA	SULMV	SULSMV	SLLMA	SLLMV	SLLSMV
Age	−0.358 ***	−0.380 ***	−0.326 ***	−0.456 ***	−0.415 ***	−0.315 ***	−0.280 ***	−0.360 ***	−0.360 ***	−0.383 ***	−0.298 ***	−0.336 ***
BMI	0.085	−0.095	0.081	−0.057	−0.076	0.064	−0.064	0.077	0.090	−0.115	0.074	0.087
ESR	−0.266 **	−0.336 ***	−0.233 *	−0.206	−0.195	−0.212 *	−0.260 **	−0.193	−0.226 *	−0.335 ***	−0.281 **	−0.274 **
HbA1c	−0.296 ***	−0.241 *	−0.21	−0.233 *	−0.218 *	−0.259 *	−0.299 **	−0.329 ***	−0.285 **	−0.182	−0.275 **	−0.236 *
TSH	−0.120	−0.214 *	−0.108	−0.106	−0.095	−0.133	−0.123	−0.100	−0.140	−0.251 **	−0.125	−0.099
Urea	−0.363 ***	−0.394 ***	−0.217 *	−0.296 ***	−0.272 **	−0.162	−0.262 ***	−0.236 **	−0.210 *	−0.387 ***	−0.384 ***	−0.344 ***

Data are represented as standardized beta coefficients. * = *p* < 0.05, ** = *p* < 0.01, *** = *p* < 0.001. SMNV—Sum of Motor Nerve Velocities, SMNA—Sum of Motor Nerve Amplitudes, SSNV—Sum of Sensory Nerve Velocities, SSNA—Sum of Sensory Nerve Amplitudes, SULSA—Sum of Upper Limb Sensory Amplitudes, SULSV—Sum of Upper Limb Sensory Velocities, SULMA—Sum of Upper Limb Motor Amplitudes, SULMV—Sum of Upper Limb Motor Velocities, SULSMV—Sum of Upper Limb Sensorimotor Velocities, SLLMA—Sum of Lower Limb Motor Amplitudes, SLLMV—Sum of Lower Limb Motor Velocities, SLLSMV—Sum of Lower Limb Sensorimotor Velocities.

**Table 4:** Multiple regression models with nerve conduction parameters serving as the dependent variable—Standardized beta coefficients.

	Age	ESR	HbA1c	TSH	Urea
SMNV	−0.217 *	−0.032	−0.356 ***	−0.181	−0.319 **
SMNA	−0.250 *	−0.137	−0.247 *	−0.193	−0.218
SSNV	−0.244 *	−0.035	−0.277 *	−0.116	−0.102
SSNA	−0.516 ***	0.001	−0.292 **	−0.092	−0.064

Data are represented as: standardized beta coefficients, * = *p* < 0.05, ** = *p* < 0.01, *** = *p* < 0.001. SMNV—Sum of Motor Nerve Velocities, SMNA—Sum of Motor Nerve Amplitudes, SSNV—Sum of Sensory Nerve Velocities, SSNA—Sum of Sensory Nerve Amplitudes, SULSA—Sum of Upper Limb Sensory Amplitudes, SULSV—Sum of Upper Limb Sensory Velocities, SULMA—Sum of Upper Limb Motor Amplitudes, SULMV—Sum of Upper Limb Motor Velocities, SULSMV—Sum of Upper Limb Sensorimotor Velocities, SLLMA—Sum of Lower Limb Motor Amplitudes, SLLMV—Sum of Lower Limb Motor Velocities, SLLSMV—Sum of Lower Limb Sensorimotor Velocities.

**Table 5:** Multiple regression models with nerve conduction parameters serving as the dependent variable—Standardized beta coefficients.

	Age	ESR	HbA1c	TSH	Urea
SULSV	−0.206	−0.018	−0.246 *	−0.084	−0.057
SULSA	−0.495 ***	0.001	−0.265 *	−0.086	−0.041
SULMV	−0.379 ***	0.071	−0.439 ***	−0.112	−0.107
SULMA	−0.235	−0.088	−0.332 ***	−0.123	−0.153
SULSMV	−0.283 *	0.011	−0.322 **	−0.114	−0.082
SLLMV	−0.070	−0.124	−0.251 *	−0.181	−0.398 ***
SLLMA	−0.245 *	−0.120	−0.157	−0.204	−0.221
SLLSMV	−0.187	−0.050	−0.286 **	−0.145	−0.309 **

Data are represented as: standardized beta coefficients, * = *p* < 0.05, ** = *p* < 0.01, *** = *p* < 0.001. SMNV—Sum of Motor Nerve Velocities, SMNA—Sum of Motor Nerve Amplitudes, SSNV—Sum of Sensory Nerve Velocities, SSNA—Sum of Sensory Nerve Amplitudes, SULSA—Sum of Upper Limb Sensory Amplitudes, SULSV—Sum of Upper Limb Sensory Velocities, SULMA—Sum of Upper Limb Motor Amplitudes, SULMV—Sum of Upper Limb Motor Velocities, SULSMV—Sum of Upper Limb Sensorimotor Velocities, SLLMA—Sum of Lower Limb Motor Amplitudes, SLLMV—Sum of Lower Limb Motor Velocities, SLLSMV—Sum of Lower Limb Sensorimotor Velocities.

## Data Availability

Datasets are available upon reasonable request from the corresponding author.
